# SUMOylation of nuclear receptor Nor1/NR4A3 coordinates microtubule cytoskeletal dynamics and stability in neuronal cells

**DOI:** 10.1186/s13578-024-01273-x

**Published:** 2024-07-13

**Authors:** Jonathan Gagnon, Véronique Caron, André Tremblay

**Affiliations:** 1 Research Center, CHU Sainte-Justine, 3175 Côte Ste-Catherine, Montréal, Québec H3T 1C5 Canada; 2 Department of Biochemistry and Molecular Medicine, Faculty of Medicine, University of Montreal, Montréal, Québec H3T 1J4 Canada; 3 Centre de Recherche en Reproduction et Fertilité, University of Montreal, Saint-Hyacinthe, Québec J2S 7C6 Canada; 4 Department of Obstetrics and Gynecology, Faculty of Medicine, University of Montreal, Montréal, Québec H3T 1J4 Canada

**Keywords:** Nuclear receptor, NR4A3, Nor-1, Neuronal protection, SUMO1, Microtubules, Cytoskeleton, Nocodazole

## Abstract

**Background:**

Nor1/NR4A3 is a member of the NR4A subfamily of nuclear receptors that play essential roles in regulating gene expression related to development, cell homeostasis and neurological functions. However, Nor1 is still considered an orphan receptor, as its natural ligand remains unclear for mediating transcriptional activation. Yet other activation signals may modulate Nor1 activity, although their precise role in the development and maintenance of the nervous system remains elusive.

**Methods:**

We used transcriptional reporter assays, gene expression profiling, protein turnover measurement, and cell growth assays to assess the functional relevance of Nor1 and SUMO-defective variants in neuronal cells. SUMO1 and SUMO2 conjugation to Nor1 were assessed by immunoprecipitation. Tubulin stability was determined by acetylation and polymerization assays, and live-cell fluorescent microscopy.

**Results:**

Here, we demonstrate that Nor1 undergoes SUMO1 conjugation at Lys-89 within a canonical ψKxE SUMOylation motif, contributing to the complex pattern of Nor1 SUMOylation, which also includes Lys-137. Disruption of Lys-89, thereby preventing SUMO1 conjugation, led to reduced Nor1 transcriptional competence and protein stability, as well as the downregulation of genes involved in cell growth and metabolism, such as *ENO3*, *EN1*, and *CFLAR*, and in microtubule cytoskeleton dynamics, including *MAP2* and *MAPT*, which resulted in reduced survival of neuronal cells. Interestingly, Lys-89 SUMOylation was potentiated in response to nocodazole, a microtubule depolymerizing drug, although this was insufficient to rescue cells from microtubule disruption despite enhanced Nor1 gene expression. Instead, Lys-89 deSUMOylation reduced the expression of microtubule-severing genes like *KATNA1*, *SPAST*, and *FIGN*, and enhanced α-tubulin cellular levels, acetylation, and microfilament organization, promoting microtubule stability and resistance to nocodazole. These effects contrasted with Lys-137 SUMOylation, suggesting distinct regulatory mechanisms based on specific Nor1 input SUMOylation signals.

**Conclusions:**

Our study provides novel insights into Nor1 transcriptional signaling competence and identifies a hierarchical mechanism whereby selective Nor1 SUMOylation may govern neuronal cytoskeleton network dynamics and resistance against microtubule disturbances, a condition strongly associated with neurodegenerative diseases.

**Supplementary Information:**

The online version contains supplementary material available at 10.1186/s13578-024-01273-x.

## Introduction

Neuron-derived orphan receptor Nor1 is a member of the NR4A subfamily of nuclear receptors, which also includes NR4A1/NGFI-B/Nur77 and NR4A2/Nurr1. NR4A receptors play pivotal roles in controlling tissue-specific gene expression pathways in development and metabolism [1–3]. Specifically, the maintenance of neuronal function and survival importantly relies on NR4A receptors in line with their dysfunction associated with neurodegenerative diseases. Rare mutations of Nurr1 have been linked to late-onset familial Parkinson's disease [4], and reduced expression levels of Nurr1, Nur77, and Nor1 were observed in brain tissues or blood of Parkinson's patients [5, 6]. Studies using Nurr1-deficient mice have shown loss of dopaminergic markers, signs of neurodegeneration, and Parkinson-like motor impairment [7, 8]. Knock-out mice lacking Nur77 also displayed disturbed dopaminergic transmission [9]. Similarly, knock-out mouse models have identified Nor1 as crucial for hippocampal pyramidal cell organization and axonal guidance during development [10] and the loss of both Nur77 and Nor1 led to abnormal myeloid cell expansion [11]. NR4A receptors have also been demonstrated to respond to increased oxidative stress [12, 13], further supporting their neuroprotective contribution to the relatively limited antioxidant defense mechanisms present in neurons during neurodegenerative diseases [14, 15]. While these observations are consistent with a neuroprotective function of NR4A receptors, the precise contribution of Nor1 and its ability to counteract disruptions in neuronal cell integrity remain elusive.

Microtubules are fundamental components of the cytoskeleton in neurons, providing dynamic structural support, enabling intracellular transport, and facilitating cellular processes such as axon growth and synaptic plasticity [16, 17]. In neurodegenerative diseases, disruptions in microtubule dynamics and integrity are associated with neuronal dysfunction and degeneration, contributing to diseases such as Alzheimer's, Parkinson's, and amyotrophic lateral sclerosis [18, 19]. Hence, microtubule-defective intracellular transport, dysregulation of microtubule-associated proteins like MAPT/Tau, alterations of tubulin modifications, and aberrant microtubule severing are commonly associated with neurodegenerative defects [20–23]. However, given the potential contribution of the NR4A receptors in neuroprotection, their role in regulating genes involved in microtubule dynamics and integrity remains unexplored.

The NR4A receptors share a very similar structure, characterized by a highly conserved DNA binding domain, enabling the receptors to interact as monomers with the NGFI-B response element (NBRE) or as homo/heterodimers with the Nur-responsive element (NurRE) of target genes [24]. In view that no bona fide endogenous ligands have as yet been identified, the transcriptional activation of NR4A receptors is thought to mainly rely on the unusual presence of bulky hydrophobic amino acids within their ligand-binding domains (LBD), which imparts them a conformation that resembles a classic activated nuclear receptor [25, 26]. Various small molecule compounds have been described to modulate NR4A activity, although their fit with the ligand-binding pocket remains uncertain [2, 27]. Also, enhanced NR4A gene expression, such as in response to growth factors, apoptotic signals, and stress, is associated with transcriptional activation [28, 29]. Therefore, there is a growing need to understand the precise mechanism by which NR4A receptors regulate transcription in various contexts and pathological conditions.

Post-translational modifications, including phosphorylation, ubiquitination, and SUMOylation, have been identified for Nurr1 and Nur77 receptors. However, very few studies have delineated the role of post-translational modifications in regulating Nor1 activity and target gene expression. Notably, DNA-dependent protein kinase has been identified as targeting Nor1 for phosphorylation [30]. More recently, we identified Lys-137 as an atypical SUMOylation site, which is under the obligate control of adjacent phosphorylation by ERK and CK2, regulating Nor1 activity and its response to disturbances in cellular redox balance [13]. These observations underscore the importance of Nor1 modifications as critical effectors in its transcriptional control in response to various cellular cues. Such regulation is also consistent with the significant role played by the ligand-independent activation function AF-1 domain in nuclear receptor-mediated gene transcription [31, 32].

In this study, we demonstrate that Nor1 is a target of SUMO1 conjugation at Lys-89 located within a canonical SUMOylation motif of the AF-1 domain of Nor1. Lys-89 SUMOylation was shown to govern Nor1 transcriptional activity, protein turnover, and target gene expression in neural cells. Also, the disruption of Lys-89 SUMOylation resulted in enhanced microtubule stability and resistance to depolymerization through a deregulated expression of microtubule severing genes. Our findings thus unveil a novel SUMO-mediated regulation of Nor1 transcriptional control, outlining a mechanism by which Nor1 SUMOylation selectively impacts microtubule cytoskeleton stability.

## Material and methods

### Plasmids

Plasmids coding for wild-type and K137R forms of Nor1 and luciferase reporter gene constructs were previously described [13]. Lys-89 mutation of Nor1 was generated by PCR mutagenesis using Pwo DNA polymerase (Roche) and verified by automated sequencing. The GFP-SUMO1 plasmid was previously described [33].

### Cell culture and treatments

Human embryonic kidney 293 cells (HEK293) and mouse hippocampal neuronal HT-22 cells were cultured in Dulbecco’s modified Eagle’s medium (DMEM; Sigma) supplemented with 5% fetal bovine serum (FBS; Wisent). Mouse neuroblastoma Neuro2a cells were maintained in DMEM supplemented with 10% FBS. Human neuroblastoma SH-SY5Y cells were grown in DMEM/F-12 (Wisent) containing 10% FBS. Transfection of cells was performed using calcium phosphate or polyethyleneimine (Gibco) as described [13, 34]. Treatments with nocodazole (1 μM-10 μM; Sigma) and MG-132 (1 μM; Sigma) were performed in DMEM.

### Lentivirus production and generation of stable cell lines

Stable human SH-SY5Y cell lines expressing wild-type or mutant forms of Nor1 were generated as previously described [13]. Briefly, parental SH-SY5Y cells were infected in the presence of Polybrene (Santa Cruz Biotechnologies) and stable clones were sorted using FACS. Lentiviral particle production was carried out in 293E cells as described [35] using pLenti-Neo-DEST-CMV-Nor1 (wt, K89R, K137R, K89,137R, and S139E) generated by recombination with the LR Clonase II (Thermo Fisher Scientific). Stable clones were sorted by FACS and validated by Western blot analysis. Control stable cells (mock) were generated as above using an empty vector.

### Luciferase assays

Luciferase assays were performed as described using NurRE_3_Luc or NBRE_3_Luc reporter constructs [13]. Luciferase activity is measured by luminescence (Nivo, Perkin-Elmer) and normalized to the β-galactosidase activity. Data are expressed as relative luciferase units (RLU) or fold response compared to control derived from at least three separate experiments performed in triplicate.

### Cell lysates and immunoblotting

Analysis of Nor1 whole cell content was performed by Western blot analysis [13] using anti-Nor1 (Santa Cruz Biotech., sc-393902), anti-HA (12CA5) or anti-GFP (Roche, 11 814 460 001) antibodies. An anti-β-actin antibody (Novus Biologicals, NB600-501) was also used to normalize total protein loading.

### Cycloheximide chase

Cycloheximide chase experiment was performed essentially as described [36]. Briefly, HT-22 cells were transfected with wild-type or K89R HA-Nor1 and harvested at the indicated time points following treatment with 50 µM cycloheximide (Sigma). Nor1 steady-state levels were analyzed by Western blot and expressed relative to β-actin content. Results were derived from at least three separate experiments.

### SUMOylation assays

SUMOylation analysis of Nor1 was determined as previously described [13]. Briefly, cells were transfected with wild-type or mutant forms of Nor1 and subjected to immunoprecipitation with an anti-HA antibody (Roche, 11 867 423 001). Western blot analysis was then performed with antibodies against Nor1 (Santa Cruz Biotech.), HA tag (12CA5), GFP (Roche), or SUMO2/3 (Abcam, ab3742) as indicated. GFP-SUMO1 plasmid was also added to transfections when indicated. Input signals were also analyzed by Western analysis of whole-cell samples.

### RNA isolation and qPCR analysis

Total cellular RNA was isolated using TRIzol reagent (Thermo Fisher Sci.), treated with DNase1 (Ambion), and subjected to reverse transcription using the RevertAid H Minus First Strand cDNA Synthesis kit (Thermo Fisher Sci.). Quantitative PCR analysis was then carried out as described [37], and values were obtained from at least three separate experiments performed in duplicate and normalized to RPLP0 gene expression.

### Real-time cell growth

Real-time analysis of cellular growth was performed using the xCELLigence RTCA Dual Purpose biosensor analyzer with the E-plate configuration (Roche) as described [13]. Briefly, stable SH-SY5Y cells were plated at 50 000 cells/well and readings were started after a pre-incubation period of 30 min at 37 °C in 5% CO_2_. Impedance magnitude recordings representative of cell number were converted to cell index (CI) values using the RTCA software 2.0 (Roche). Background readings were performed with medium alone. Values are derived from at least three separate experiments performed in duplicate.

### MTT viability assay

Cell viability was assessed using the 3-(4,5-dimethylthiazol-2-yl)-2,5-diphenyl tetrazolium bromide (MTT; Sigma) assay as described [38]. Briefly, cells were seeded in 24-well plates at a density of 100 000 cells/well. At day 0, cells were treated with 1 μM nocodazole for 6 h or vehicle (control) and 0.5 mg/ml MTT was then added for 2–3 h prior to extraction with acidified isopropanol. Absorbance was read at 570 nm and 690 nm (non-specific) by spectrophotometry. Results are derived from at least three independent experiments performed in triplicate.

### Analysis of polymerized and acetylated tubulin

Polymerized microtubules and tubulin acetylation were assessed as previously described with modifications [39]. Briefly, SH-SY5Y stable cells were treated or not (vehicle) with 1 μM nocodazole for 4 h at 37 °C, washed in PBS and then lyzed in Pipes (50 mM, pH 6.9) buffer containing 50 mM NaCl, 5 mM MgCl_2_, 5 mM EDTA, 5% glycerol, 0.1% Igepal, 0.1% Triton X-100, 0.1% Tween-20, 0.1% β-mercaptoethanol, 1 mM ATP, 0.1 mM GTP and protease inhibitors (Roche) to preserve microtubule integrity. Whole-cell lysates were used for analysis of acetylated tubulin. To prepare the microtubule fractions, cells were lyzed in the same conditions and lysates were then clarified by centrifugation to isolate the supernatant which was used for measurement of soluble tubulin (globular fraction). The pellet representing the assembled microtubule (filamentous fraction). The pellet was resuspended in RIPA buffer supplemented with 1% Igepal, 0.05% sodium deoxycholate, 0.1% SDS, 1 mM PMSF, and protease inhibitors (Roche). Samples were proportionally loaded and analyzed by immunoblotting using antibodies for α-tubulin (Santa Cruz Biotech., sc-134239). Acetylation of tubulin was assessed using an antibody for acetylated Lys-40 tubulin (Sigma, T-7451) and a pan-tubulin antibody (Abcam, ab11304) was used to monitor loading.

### Live-cell fluorescent microscopy

The relative microtubule stability in the context of Nor1 expression was determined by fluorescent microscopy with the use of YFP-Nor1 (WT, K89R, K137R) fusion constructs [13] and mCherry-TUBA1 expression plasmid based on described procedures [40–42]. To generate the pCMX-mCherry-TUBA1 construct, the human α-tubulin coding region was amplified and inserted in frame at the C-terminal end of the mCherry coding insert kindly provided by Rubén Marín Juez. HT-22 cells were transfected using polyethylenimine (Gibco) and cultured on poly-D-lysine coated coverslips (Sigma-Aldrich). Cells were treated with 1 μM nocodazole or vehicle (DMSO; 1/1,000, v/v) for 4 h and then processed for real-time fluorescence analysis using a Leica DMi8 microscope and the Application Suite LAS X software. ImageJ (v2.14) software was used to quantify relative protein levels of Nor1 (YFP channel) and TUBA1 (mCherry channel) expressed as integrated density normalized to the area coverage in μm^2^ and to background measured from a cell-free region in the same field of view. Each independent channel was used to determine the protein levels of interest per each cell transposed to mCherry/YFP ratio (mean ± SEM) for untreated and nocodazole-treated cells. The same approach was also performed in the context of the K40R and K40Q variants of TUBA1 generated by PCR mutagenesis and using the mCherry-actin-7 plasmid from Addgene (#54966). All measurements were made from 16-bit raw images obtained from at least three independent experiments performed in duplicate.

### Statistics

Statistical comparisons for three groups or more were determined by one-way ANOVA followed by Bonferroni post-hoc analysis. Single comparisons between two groups were determined using unpaired two-tailed Student’s t-test performed with the GraphPad Prism software. Values are presented as mean ± SEM from at least three separate experiments performed in duplicate or otherwise stated. P values smaller than 0.05 were considered statistically significant.

## Results

### Nor1 is a target of SUMO1 conjugation at Lys-89 contained in a consensus SUMOylation motif

**Fig. 1:**
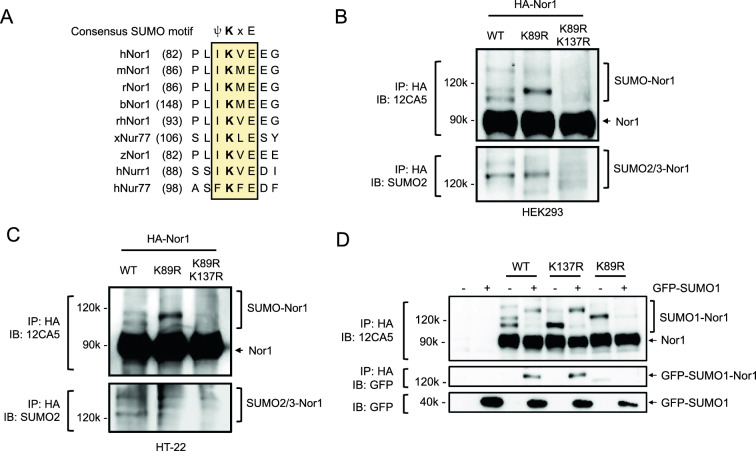
Nor1 is SUMOylated by SUMO1 at Lys-89. **A** Homology sequence alignment of the N-terminal canonical SUMOylation motif ψKxE between Nor1-expressing species and members of the NR4A subfamily. The number in parenthesis represents the position of the first amino acid in the sequence. Conserved residues of the motif are shaded. h, human; m, mouse; r, rat; b, bos taurus; rh, rhesus monkey; x, xenopus; z, zebrafish. **B** SUMOylation of Nor1 at Lys-89 and Lys-137. SUMOylation assay was performed in 293 cells transfected with HA-tagged wild-type or lysine-to-arginine mutants of Nor1. Samples were immunoprecipitated with an anti-HA antibody, and Western analysis was performed using 12CA5 and anti-SUMO2 antibodies. **C** Same as in B, except that SUMOylation assay was performed in HT-22 neuronal cells. **D** SUMOylation assay was performed in 293 cells transfected with HA-tagged wild-type or SUMO-mutants of Nor1 in the presence or absence of GFP-tagged SUMO1. Anti-HA antibody was used for immunoprecipitation, and Western analysis was performed with 12CA5 and anti-GFP antibodies

Our previous work has described a non-consensus SUMOylation motif, referred to as the pSuM. which allows the conjugation of poly-SUMO2 at Lys-137 of Nor1 [13]. We also observed that mutating Lys-137 did not completely abrogate Nor1 SUMOylation, highly suggesting that additional SUMO conjugation may occur on Nor1. Interestingly, our sequence homology analysis using the ψKxE sequence, which is considered the classic consensus SUMOylation motif [43], has revealed that Lys-89 is part of a ψKxE motif located in the AF-1 region of Nor1. This ψKxE motif is perfectly conserved across vertebrates and lower species and among the other members of the NR4A subfamily, strongly supporting a functional role in Nor1 function (Fig. 1A). We thus analyzed the SUMOylation potential of Nor1 in human embryonic kidney 293 cells and found a distinctive pattern of Nor1 SUMOylation, characterized by the presence of three bands of respective molecular weights of ~ 100 kDa, ~ 114 kDa, and ~ 135 kDa (Fig. 1B). The same pattern of SUMOylation was also found using murine neuronal hippocampal HT-22 cells (Fig. 1C), which may support a shared pattern of Nor1 SUMOylation in different cell types. Based on our previous findings also describing a three-band pattern of Nor1 SUMOylation, we identified the middle and highest bands to be associated with Lys-137 SUMO2/3 conjugation [13]. Interestingly, mutation of Nor1 Lys-89 resulted in the complete disappearance of the lower band at ~ 100 kDa, which was also not recognized by a SUMO2 antibody (Fig. 1B and C). This suggests that the ~ 100 kDa band might represent an additional SUMOylation site not related to SUMO2/3. Also, mutating Lys-89 and Lys-137 together (K89,137R mutant) abolished the three distinctive bands, supporting the role of Lys-89 and Lys-137 as major SUMOylation sites of Nor1. To ascertain whether Lys-89 could conjugate SUMO1, we then tested the effect of expressing GFP-SUMO1 in cells and found that mutating Lys-89 abolished the upshift in SUMO1 signal of Nor1 (Fig. 1D). Conversely, mutation at Lys-137 did not impair Nor1 SUMO1 conjugation. These results support Lys-89 as a target of SUMO1. Intriguingly, we also noticed that disrupting either Lys-89 or Lys-137 was affecting the intensity of each other SUMO signals compared to wild-type receptor. Indeed, each signal corresponding to either Lys-89 (lower ~ 100 kDa band) or to Lys-137 SUMOylation (~ 114 kDa) was found increased in intensity upon the disruption of the other respective site (Fig. 1D). Whether this might reflect a cross-regulation mechanism between the two SUMOylation sites is possible but remains to be ascertain.

### Lys-89 SUMOylation promotes Nor1 transcriptional activity and target gene expression

**Fig. 2:**
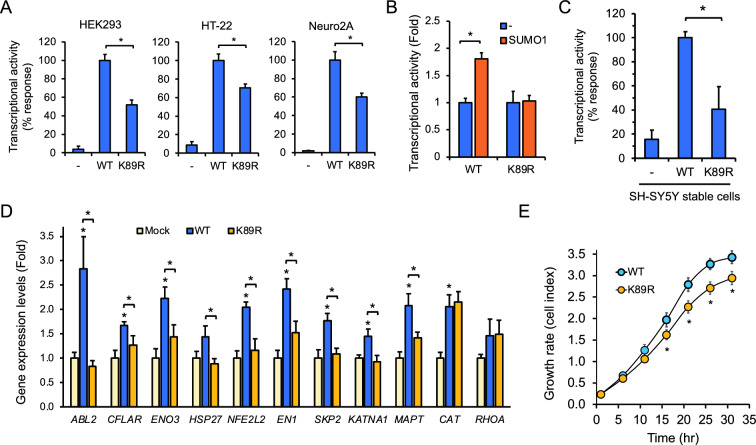
SUMOylation at Lys-89 induces Nor1 transactivation and target gene expression in neural cells. **A** Disruption of Lys-89 SUMOylation impairs Nor1 transcriptional activity in 293, HT-22, and Neuro2A cells. Cells were transfected with wild-type or K89R-mutated Nor1 in the presence of the NurRE_3_-Luc reporter and harvested for luciferase activity measurement. Values are shown as mean ± SEM in percentage response compared to wild-type cells set at 100%. Data are derived from at least three separate experiments done in triplicate. *P < 0.001 vs. control. **B** Luciferase assay in 293 cells transfected with wild-type Nor1 or K89R mutant in the presence of the NurRE_3_-Luc reporter and SUMO1. Values are derived from at least three independent experiments realized in triplicate and expressed as fold (mean ± SEM) compared to control cells set at 1.0. *P < 0.01 vs. control. **C** Luciferase assay was performed in human neuroblastoma SH-SY5Y cells stably expressing wild-type Nor1 or K89R mutated Nor1 and compared to mock-transfected cells (-). The NurRE_3_-Luc reporter was used in transfection, and cells were harvested for determination of luciferase activity. Values were normalized to β-galactosidase activity and are expressed (mean ± SEM) as a percentage relative to wild-type Nor1-expressing cells set at 100%. Values are derived from at least three independent experiments repeated in triplicate. *P < 0.05 vs. control. **D** qPCR analysis was performed in the stable SH-SY5Y cell lines. Gene expression levels were derived from at least three separate experiments and normalized to RPLP0 expression. Results are expressed as fold response (mean ± SEM) relative to mock control cells set to 1.0 for each gene. *P < 0.05. **E** Disruption of Nor1 Lys-89 SUMOylation impedes neuronal cell growth. SH-SY5Y stable cells were seeded and monitored for cell proliferation using impedance measurement for each indicated time. Results are expressed as cell index (mean ± SEM) derived from at least three independent experiments performed in duplicate. *P < 0.05

We next addressed the effect of Lys-89 SUMOylation on Nor1 transcriptional activity using the NurRE_3_-Luc reporter in luciferase reporter assay. We found that the K89R mutant exhibited a significant decrease in activity when compared to wild-type Nor1 in 293 cells, with similar results also obtained in neuronal HT-22 and Neuro2A cells (Fig. 2A). To determine whether SUMO1 could contribute to Nor1 activation involving Lys-89 SUMOylation, ectopic expression of SUMO1 led to an increase in wild-type Nor1 activity, whereas the K89R mutant remained mostly unaffected (Fig. 2B). These results indicate that Lys-89 SUMO1 conjugation can promote Nor1 transcriptional activation**.**

To gain more insights into the positive role of Lys-89 SUMOylation on Nor1 activity, we generated stable neuroblastoma SH-SY5Y cell lines expressing either wild-type Nor1 or the K89R mutant. Again, K89R-expressing cells showed an impaired transcriptional response compared to wild-type Nor1 cells (Fig. 2C). To support Lys-89 dependent activation of Nor1, we next analyzed the expression levels of Nor1 regulated genes in SH-SY5Y cell clones. We found that Nor1-responsive genes, such as *ABL2, CFLAR*, *ENO3*, *EN1*, and *SKP2*, were significantly decreased in K89R-expressing cells compared to wild-type Nor1 (Fig. 2D). Interestingly, other responsive genes such as *CAT* that was identified as a Nor1 regulated gene under conditions of oxidative stress, as well as *RHOA* [13] were not downregulated in K89R-expressing stable cells, supporting a gene-selective regulation by Nor1 Lys-89 SUMOylation. It is also of interest to note that *KATNA1* and *MAPT*, two genes related to microtubule cytoskeleton dynamics, were also found upregulated by Nor1 in a Lys-89 dependent fashion (Fig. 2D). Similar results on the effect of the K89R mutation on Nor1 regulated gene levels were observed in mouse neuronal HT-22 cells (Fig. S1), further supporting the gene-selective response to Nor1 Lys-89 SUMOylation. Concurring with the downregulation of Nor1 responsive genes associated with cell proliferation, like *SKP2, ATF3, and ENO3* in K89R stable cells, we next addressed whether Nor1 Lys-89 SUMOylation could affect cell growth. Real-time monitoring of cell growth was determined by impedance analysis of stable cells maintained in culture. We found that the proliferation of Nor1 K89R-expressing SH-SY5Y cells was significantly slower compared to wild-type Nor1 cells (Fig. 2E). These results specify a role of Lys-89 SUMOylation to promote Nor1-dependent gene transcription and growth of neural cells.

### SUMOylation at Lys-89 affects Nor1 protein stability

**Fig. 3:**
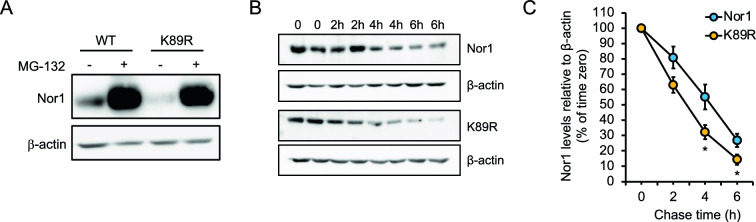
SUMOylation at Lys-89 increases Nor1 protein stability**. A** 293 cells were transfected with HA-tagged wild-type, or K89R mutated Nor1 and then treated or not with 1 μM proteasome inhibitor MG-132. Western blot analysis of Nor1 protein levels was performed with an anti-HA antibody, and β-actin was used to monitor protein loading. **B** Cycloheximide chase experiment in HT-22 cells transfected with HA-tagged wild-type or mutant Nor1. Cells were treated with 50 µM cycloheximide and harvested at the indicated times after treatment. Western blot analysis was performed using an anti-HA antibody, and β-actin was used to monitor protein loading. Representative blots of experimental duplicates are shown. **C** Signal intensities from three independent experiments carried out as in B were quantified and normalized to β-actin levels. Values are represented as a percentage (mean ± SEM) of time zero, which is set at 100%. *P < 0.05 vs. wild-type

We next addressed the role of Lys-89 in affecting Nor1 protein turnover. Protein levels of wild-type Nor1 were greatly increased in response to the 26S proteasome inhibitor MG-132, indicating that Nor1 is subjected to proteasomal degradation (Fig. 3A). Similarly, Nor1 K89R variant levels were also increased by MG-132. However, the steady-state levels of K89R in absence of MG-132 were highly diminished compared to wild-type Nor1, suggesting that Lys-89 is important for Nor1 stability in cells. We thus performed cycloheximide chase experiments to address whether Lys-89 could affect Nor1 protein turnover and measured a more rapid protein turnover of the K89R mutant (τ½ = 2.8 h ± 0.3) compared to wild-type Nor1 (τ½ = 4.4 h ± 0.4; P = 0.032; Fig. 3B and C). These results indicate a role of Lys-89 SUMOylation in stabilizing Nor1 protein.

### Microtubule disruption increases Nor1 gene expression and Lys-89 SUMOylation

**Fig. 4:**
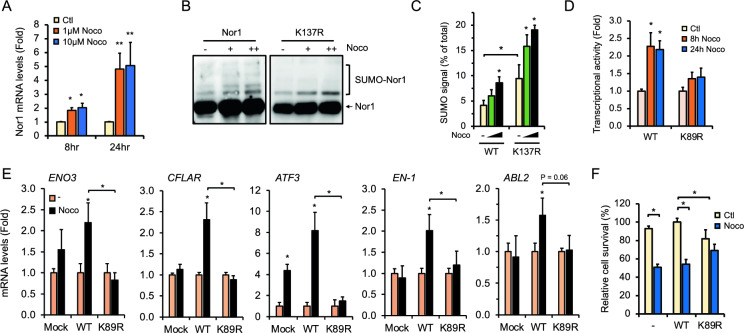
Lys-89 SUMOylation regulates Nor1-responsive genes and neural cell survival to microtubule disorganization. **A** Microtubule disruption induces Nor1 gene expression. qPCR analysis was performed on HT-22 cells treated with nocodazole or vehicle (Ctl) with the indicated concentrations and time periods. Results were normalized to RPLP0 expression and expressed (mean ± SEM) as fold response relative to untreated cells set to 1.0. *P < 0.05; **P < 0.01 vs. control. **B** Nor1 SUMOylation is increased by nocodazole. SUMOylation assay was performed in HT-22 cells transfected with HA-tagged wild-type or K137R mutant Nor1 treated or not (vehicle) with 1 or 10 μM of nocodazole for 24 h. **C** Measurement of signal intensities of Lys-89 specific SUMOylation derived from three separate experiments performed as in (**B**). Values are expressed as the percentage of Lys-89 SUMOylation intensity (mean ± SEM) compared to total Nor1. *P < 0.05. **D** Transcriptional assay was performed in HT-22 cells transfected with wild-type Nor1, or K89R mutated Nor1 in the presence of the NBRE_3_-Luc reporter. Cells were treated with 10 μM nocodazole or not (vehicle) for the indicated time. Luciferase values were normalized to β-galactosidase activity and expressed (mean ± SEM) as a fold response relative to control cells set at 1.0. Values are derived from at least three independent experiments repeated in triplicate. *P < 0.05 vs. control. **E** Induction of Nor1-responsive genes by nocodazole. qPCR analysis of SH-SY5Y stably expressing wt or K89R Nor1. Cells were treated or not (vehicle) with 1 μM nocodazole. Values are presented as fold response (mean ± SEM) compared to untreated controls set to 1.0 for each cell line. Values were normalized to RPLP0 gene expression. *P < 0.05. **F** MTT cell viability assay was performed in HT-22 cells expressing wt or K89R Nor1. Cells were treated or not (vehicle) with 1 μM nocodazole for 6 h. Values are presented as mean ± SEM of percent change compared to untreated wt Nor1 cells set at 100%. Results are derived from at least three separate experiments. *P < 0.05

Microtubules are a critical component of healthy neurons, and the balance between microtubule dynamics and stability is essential for neuronal maintenance and plasticity of the nervous system [44, 45]. Hence, anomalies in microtubule integrity are strongly associated with neurodevelopmental and neurodegenerative diseases [18, 19]. However, not much is known about Nor1's contribution to the microtubule network. Our findings that microtubule-associated genes such as *KATNA1* and *MAPT* were upregulated by Nor1 and Lys-89 SUMOylation (Fig. 2B) highly support a functional role of Nor1 in regulating microtubule dynamics. We thus treated neuronal HT-22 cells in a dose- and time-dependent fashion with nocodazole, a potent disrupting drug of microtubule polymerization. Under these conditions, Nor1 mRNA expression was strongly enhanced, suggesting a positive gene regulation of Nor1 in response to microtubule disturbances (Fig. 4A). Also, Nor1 SUMOylation was increased by nocodazole with a significant induction of the Lys-89 SUMOylation signal as determined in the context of wild-type Nor1 and the K137R mutant (Fig. 4B, C). In addition, nocodazole treatment of cells resulted in an increase in Nor1 transcriptional activity in a manner dependent on Lys-89 SUMOylation (Fig. 4D). These results imply a functional transcriptional response of Nor1 to microtubule disruption by nocodazole in neuronal cells.

To further support such transcriptional regulation, we found that several Nor1-responsive genes identified to be sensitive to Lys-89 SUMOylation (Fig. 2D and Fig. S1), such as *ENO3*, *CFLAR*, *ATF3*, and *EN-1*, were significantly upregulated in response to nocodazole in wt Nor1-expressing cells (Fig. 4E). However, such regulation was prevented in the context of K89R mutant expressing cells, correlating with a role of Lys-89 SUMOylation to enhance Nor1 activity in conditions of microtubule disorganization. Given the potential of these genes to regulate cell growth and metabolism, we next assessed whether Nor1 SUMOylation at Lys-89 could impact cell survival in response to the nocodazole insult. As expected, nocodazole treatment led to a significant decrease in cell survival rates in both control and Nor1-expressing HT-22 cells compared to each respective untreated control (Fig. 4F). However, K89R expressing cells were found to be more resistant to nocodazole with no significant changes in cell survival rates compared to control cells. A similar protective effect of the K89R mutant was also observed in the context of neuro-2A and SH-SY5Y cells (Fig. S2A and B). These results were rather intriguing given the effect of K89R mutation to impair cell growth in the absence of nocodazole but raise a possible role of Nor1 Lys-89 SUMOylation in the detrimental response of neuronal cells to microtubule disorganization induced by nocodazole.

### Divergent role of Lys-89 and Lys-137 SUMOylation in Nor1 regulation of microtubule organization genes and stabilization

**Fig. 5:**
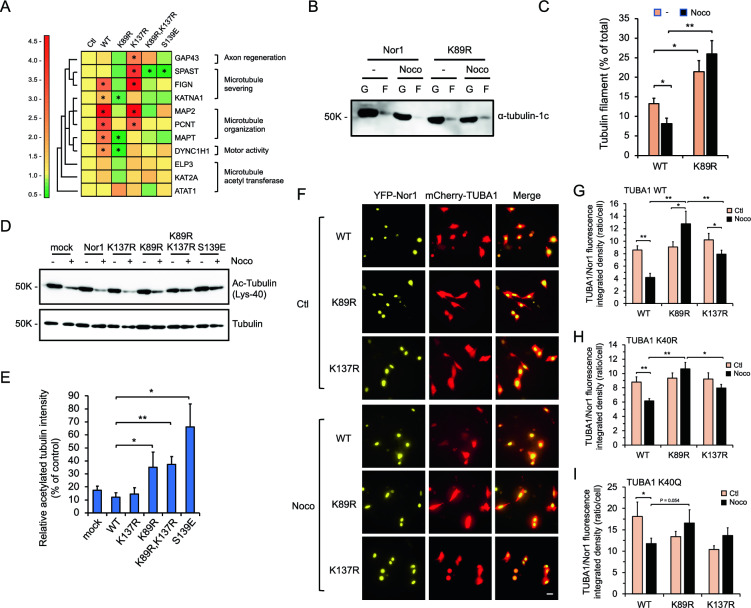
Divergent role of Lys-89 and Lys-137 SUMOylation in Nor1 regulation of microtubule organization genes and stabilization in neural cells. **A** Heat map representation and cluster analysis of expression levels of genes associated with microtubule dynamics and stability in SH-SY5Y stable cell lines treated or not (vehicle) with 1 μM nocodazole. Values are presented as fold response (mean ± SEM) compared to respective untreated controls set to 1.0 for each cell line. Data were derived from at least three independent experiments and normalized to RPLP0 gene expression. *P < 0.05 vs untreated controls. **B** Analysis of microtubule (filamentous tubulin; F) proportion compared to free tubulin (globular tubulin; G) in response to nocodazole in the SH-SY5Y cell lines treated or not (vehicle) with 1 μM nocodazole for 4 h. Western blot analysis was carried out using an anti-α-tubulin-1c antibody. **C** Intensity quantification from three separate experiments was carried out as in B. The ratio of filamentous tubulin (F) to globular tubulin (G) was determined, and values are expressed as mean percent ± SEM of filamentous tubulin. *P < 0.01, **P < 0.001 compared to untreated cells. **D** Tubulin acetylation in SH-SY5Y stable cell lines expressing wt or variants of Nor1 as indicated. Cells were treated or not (vehicle) with 1 μM nocodazole for 4 h and assessed by Western blot analysis using an anti-acetylated Lys-40 tubulin antibody. A pan tubulin antibody was used to monitor control loading. **E** Signal intensity quantification from at least three separate experiments performed as in C. Results were normalized using anti-tubulin antibody and are expressed as a percentage (mean ± SEM) of acetylated tubulin left after nocodazole treatment relative to untreated controls set at 100% for each cell line. *, P < 0.05, **; P < 0.005. **F** Mouse HT-22 neuronal cells were transfected with YFP fusions of WT, K89R or K137R Nor1 variant in the presence of mCherry-TUBA1 expression plasmid. Cells were then treated or not with 1 µM nocodazole for 4 h and visualized in real-time by fluorescent microscopy. Representative fluorescent microscopy images are shown depicting the protein levels of Nor1 and α-Tubulin in each condition. Scale bar: 25 μm. **G** Quantitation of red (Cherry) fluorescence was determined and normalized to yellow (YFP) fluorescence per each single cell analyzed. Values are derived from three independent experiments performed as in F and presented as mean ± SEM of TUBA1 to Nor1 ratios in untreated (vehicle) control (Ctl) and nocodazole-treated (Noco) cells. *P < 0.05; **P < 0.001. **H** Similar as in G except that the acetylation-defective mCherry-TUBA1 K40R construct was used in cells. *P < 0.05; **P < 0.001. **I** Similar as in G except that the acetylation-mimic variant mCherry-TUBA1 K40Q construct was used in cells. *P < 0.05

Our findings on the regulation of *MAPT* and *KATNA1* genes by Nor1 and Lys-89 SUMOylation (Fig. 2B and S1) strongly suggest a role of Nor1 in microtubule dynamics and reorganization in neuronal cells. To better understand how Lys-89 may contribute to microtubule organization, we thus analyzed the expression of key genes involved in microtubule networking and dynamics in stably expressing SH-SY5Y cells. Interestingly, several genes associated with microtubule stability and organization were found upregulated in wt Nor1 cells in response to nocodazole, whereas such regulation was either impaired or even reversed in K89R stable cells (Fig. 5A). Most notably, genes involved in the severing of microtubules, such as *KATNA1*, *SPAST*, and *FIGN* were remarkably down-regulated by nocodazole treatment in K89R stable cells compared to wt Nor1 cells, implying that Nor1 Lys-89 integrity was needed to achieve proper gene response to microtubule disruption. A similar downregulation in microtubule severing gene expression was also observed in the context of the S139E variant, which is known to constitutively induce Lys-137 SUMOylation and reduce Nor1 activity [13], whereas the SUMO-defective K137R variant had the opposite effect (Fig. 5A). Nor1 Lys-137 was described as embedded in a pSuM motif, dictating SUMO2 recruitment and transcriptional repression under the phosphorylation of Ser-139 required to provide the obligate negative charge for effective SUMO conjugation [13]. These results are thus consistent with a dual role of Lys-89 and Lys-137 SUMOylation in Nor1 regulation of severing genes in conditions of microtubule disorganization. Among other microtubule-related genes that follow such apparent SUMO-specific regulation by Nor1 were genes involved in microtubule cytoskeleton organization, such as *MAPT*, *MAP2*, *PCNT*, and in motor activity like *DYNC1H1* (Fig. 5A). Conversely, the regulation of *ATAT1* gene involved in tubulin acetylation appears to diverge in the other way, showing an increase in expression levels in K89R stable cells compared to wt or K137R Nor1 cells.

To further address the functional impact of Nor1 Lys-89 SUMOylation on the microtubule network, we next measured the proportion of α-tubulin assembled in microtubules (filamentous fraction; F) relative to free α-tubulin (globular fraction; G) as an indicator of microtubule organization. We found that in absence of nocodazole, K89R stable cells already exhibit a greater ratio of α-tubulin filaments over soluble α-tubulin (F/F + G) relative to wt Nor1-expressing cells, suggestive of enhanced microtubule polymerization in K89R stable cells (Fig. 5B, C). As expected, nocodazole treatment significantly decreased the α-tubulin filamentous fraction in wt Nor1 cells, whereas no such effect was observed in K89R stable cells. This might indicate a less protective effect of cells to nocodazole in conditions of Nor1 Lys-89 SUMOylation. To further support the impact of Lys-89 SUMOylation on microtubule stabilization, we measured the levels of acetylated Lys-40 tubulin, a marker of long-lived microtubule mechanical stability [46, 47]. We found a significant increase in acetylated tubulin levels in nocodazole-treated K89R stable cells compared to wild-type Nor1-expressing cells, again indicating a less protective effect of Nor1 Lys-89 SUMOylation to the nocodazole stress (Fig. 5D, E). When addressing the role of Lys-137 SUMOylation, we found no significant changes in tubulin acetylation levels in pSuM-defective K137R-expressing SH-SY5Y cells when compared to wt Nor1 cells in response to nocodazole (Fig. 5D, E). However, the S139E variant greatly enhanced the tubulin acetylation levels in SH-SY5Y cells, in support of a strong protective role of Nor1 Lys-137 SUMOylation to the nocodazole insult. As such, the protective effect of Lys-137 SUMOylation appears in contrast with Lys-89 SUMOylation, inferring a divergent contribution of both SUMOylation sites in Nor1 regulation of microtubule stabilization. In addition, the effect of the double K89,137R mutant was similar to that of the K89R mutation, possibly emphasizing a stronger impact of Lys-89 modification on regulating tubulin acetylation.

Tubulin subunits are known to unfold and degrade upon severing, supporting the role of microtubule severing in tubulin protein turnover [21]. Based on the effect of Nor1 Lys-89 SUMOylation on severing gene expression and tubulin dynamics, we next investigated whether α-tubulin content was affected in live cells expressing Nor1 or SUMO-defective variants using a dual-probe fluorescence microscopy approach. As expected, Nor1 was found predominantly, if not strictly, expressed in the nucleus of HT-22 cells, while α-tubulin was found in both the cytosolic and nuclear compartments, along with its role in cytoskeletal organization and cell division [48, 49]. Consistent with its known effect on tubulin depolymerization, nocodazole treatment contributed to a decrease in the TUBA1 single-cell ratios relative to Nor1 expression, indicative of tubulin degradation (Fig. 5F, G). However, these effects were not observed and even reversed in K89R-expressing cells, while K137R-expressing cells showed a reduced cellular tubulin content similar to that of wild-type Nor1-expressing cells. These findings are consistent with a protective role of Nor1 K89R against the nocodazole depolymerization insult. As a control, wild-type Nor1 or SUMO-defective variants had no effect on cellular actin levels under the same conditions (Fig. S3).

Given that K89R-expressing cells exhibited enhanced levels of acetylated α-tubulin (Fig. 5E), we next performed the same approach using the acetylation-defective K40R mutant of TUBA1. Interestingly, K89R-expressing cells still showed greater levels of TUBA1 K40R to nocodazole treatment compared to wild-type Nor1 and to K137R variant (Fig. 5H). However, the extent to which K89R raised non-acetylated TUBA1 levels was not as high as those of wild-type TUBA1 (Fig. 5G), possibly indicating another mechanism besides tubulin acetylation that may participate in the protective effect promoted by the Nor1 K89R variant. Similar results were obtained using the K40Q mutant of TUBA1, known to mimic Lys-40 acetylation. Despite higher overall expression levels of the TUBA1 variant, probably reflecting its constitutive acetyl-mimic nature, we again found a higher proportion of the K40Q mutant in K89R-expressing cells compared to wild-type or K137R-expressing cells in the context of nocodazole treatment (Fig. 5I).

**Fig. 6:**
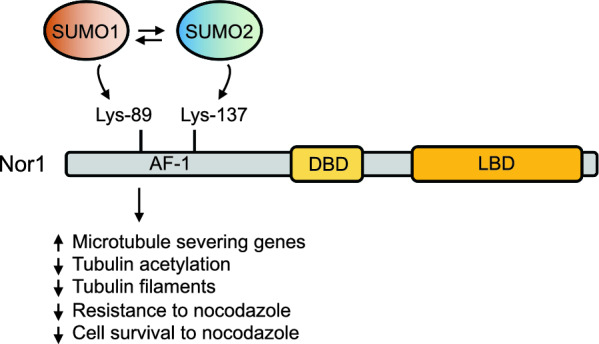
Proposed model illustrating the adaptive effects controlled by Nor1 SUMOylation on microtubule dynamics and resistance to perturbations in neuronal cells. SUMO1 conjugation occurs at Lys-89, inducing Nor1 transcriptional activity and the expression of target genes involved in cell survival and microtubule dynamics. In conditions of microtubule disturbances induced by nocodazole, Lys-89 SUMOylation appears insufficient to confer microtubule stability and resistance, resulting in increased levels of microtubule severing genes and reduced α-tubulin acetylation and microfilament organization. However, these effects appear to contrast with Lys-137 SUMOylation, thereby outlining a hierarchical mechanism of Nor1 activation based on distinct input SUMOylation signals

Taken together and as illustrated in Fig. 6, these results provide a cellular mechanism as to how Nor1 distinctive SUMOylation might dictate the transcriptional regulation of specific microtubule-related genes in the protection of neuronal cells from microtubule disorganization.

## Discussion

Characterizing NR4A receptors is a challenging task complicated by their atypical ligand-binding domain and the absence of a clearly defined endogenous ligand. Hence, identifying other regulating events that target NR4A receptors could significantly enhance our understanding of their function in both normal and pathological conditions, such as neurodegenerative disorders. In this study, we present evidence that Nor1 undergoes SUMO1 conjugation at Lys-89, located in the AF-1 domain. This SUMOylation of Nor1 enhances its stability and transcriptional potential, resulting in elevated target gene expression and increased proliferation of neuronal cells. Moreover, our findings reveal that the disruption of Lys-89 SUMOylation impairs the expression of several microtubule-related genes, thereby delineating the pivotal role of Nor1 and its SUMO conjugation in regulating microtubule dynamics.

We have established that Nor1 is subjected to SUMO1 conjugation at Lys-89, which is contained in a canonical SUMOylation ψKxE motif highly conserved across species and shared with the other two NR4A family members. Disruption of Lys-89 led to reduced Nor1 transcriptional activity and decreased expression of several Nor1-responsive genes, including *ENO3*, *EN1*, and *SKP2*, supporting a transactivating potential for Lys-89 SUMOylation. Additionally, Lys-89 SUMOylation enhanced Nor1 stability in a proteasome-dependent manner, contributing to improved Nor1 function. This contrasts with Nurr1, in which the corresponding Lys-91 was reported to conjugate SUMO2, resulting in transcriptional repression [50]. Lys-104 of Nur77 was also identified as a target of SUMO2/3, although its specific impact on Nur77 has only been studied in conjunction with Lys-555 and Lys-577 disruption, which resulted in enhanced Nur77 activity [51, 52]. Overall, this suggests that SUMOylation of NR4A receptors can lead to divergent effects on their activity. However, whether this difference in activity relates to SUMO isoform-specific conjugation of NR4A members or is influenced by various cellular contexts and reporter systems remains unclear, but it further exemplifies the broad spectrum of effects associated with target and site-specific SUMOylation [53, 54]. Consistent with this, we also reported that Lys-137 of Nor1 is an essential site for SUMO2/3 conjugation, a process tightly regulated by phosphorylation-mediated events and linked to transcriptional repression of the receptor [13]. Such divergent transcriptional impacts of conjugating SUMO1 to Lys-89 and SUMO2/3 to Lys-137 underscore the intricate and highly regulated nature of the SUMOylation process in governing Nor1-dependent gene regulation in neuronal cells.

Our data strongly suggest that SUMOylation at Lys-89 significantly contributes to neuronal cell proliferation by Nor1. In line with this, several genes associated with cellular metabolism and growth, including *ENO3, CFLAR, EN-1, SKP2*, and *ATF3*, were responsive to Nor1-enhanced transcriptional activity. This regulation is likely mediated through the direct recruitment of Nor1 to promoter regions, as NBRE elements have been identified in genes like *ENO3, CFLAR*, and *ATF3* and described to direct their transcriptional regulation through enhanced Nor1 binding and activating histone marks [13]. Notably, these genes also exhibited increased expression in nocodazole-treated cells, suggesting their responsiveness to microtubule depolymerization. Genes such as *ENO3, SKP2, EN1, CFLAR*, and *ATF3* play diverse roles in neuronal development, differentiation, energy metabolism, and anti-apoptotic effects. For instance, ATF3 is often induced in the nervous system in response to various stressors such as nerve injury, ischemia, or inflammation. ENO3 is also linked to neuroprotection in response to cellular oxidative stress [13, 55, 56]. The increased expression of Nor1-responsive genes to the nocodazole insult could therefore be seen as an attempt to rescue neuronal cells from microtubule disturbances. Furthermore, our observation that these effects were blunted in K89R-expressing cells, leading to reduced cell growth, suggests that Lys-89 SUMOylation may serve to relay neuronal survival signals by transcriptionally upregulating the expression of key genes involved in neuronal cell function and growth.

Maintaining microtubule integrity is crucial for ensuring the health of neurons, and any disruptions in microtubule equilibrium are hallmark features of many neurodevelopmental and neurodegenerative diseases [19, 45]. However, very little is known about the role of NR4A receptors in regulating genes associated with the microtubule cytoskeleton and reorganization. Here we show that Nor1 gene expression is induced under conditions of microtubule disruption. This was accompanied by an increase in several genes involved in microtubule dynamics and stability. Such upregulation of Nor1 gene expression aligns with the protective cellular response often associated with increased NR4A gene expression to various stress-inducing factors [29, 57, 58]. Enhancement of Nor1 expression, along with its transcriptional activation through Lys-89 SUMOylation, may thus support a functional role of Nor1 in maintaining the delicate balance of microtubules in neuronal cells.

As part of the mechanistic response of Nor1 to microtubule disorganization, we found that nocodazole promoted Nor1 Lys-89 SUMOylation, thereby facilitating its transcriptional activation. However, the precise mechanism underlying Nor1 SUMOylation remains unclear. Previous studies have reported that depolymerizing microtubules with nocodazole or colchicine led to the activation of kinase signaling pathways, including MAPK/Erk members, among others, to restore microtubule growth and rescue [59–61]. Interestingly, activation of the MAPK pathway is essential for promoting Nor1 SUMOylation at Lys-137, suggesting that additional regulatory mechanisms for Lys-89 SUMOylation might also take place [13]. In line with this, we reported that the S139A mutation, which disrupted Lys-137 pSuM SUMOylation, also impacted the remaining Nor1 SUMOylation signal, which might infer a role of the MAPK-directed pSuM phosphorylation in potentially coordinating distant SUMOylation at Lys-89 [13]. Further studies will be required to elucidate the intrinsic mechanism of site-specific SUMOylation controlling the overall function of Nor1.

Our observation that Nor1 K89R variant conferred better survival of neural cells in response to nocodazole implies that Lys-89-dependent dysregulation of Nor1 activity significantly impacts microtubule stabilization and resistance to nocodazole-induced depolymerization. Consistent with this, we identified Nor1 as a transcriptional regulator of critical genes associated with microtubule dynamics. Specifically, genes related to microtubule severing, such as *KATNA1* and *FIGN*, as well as genes involved in microtubule organization, such as *MAPT, MAP2,* and *PCNT*, were highly upregulated in Nor1-expressing cells, while these effects were either abolished or even reversed in K89R mutant cells. Katanin, fidgetin, and spastin are enzymes responsible for microtubule cutting, promoting the loss of microtubule integrity, and their aberrant regulation is strongly associated with neurodegenerative anomalies [62–64]. Also, through combined single-molecule fluorescence and electron microscopy, katanin and spastin were shown to remove tubulin dimers from microtubule lattices, creating sites of damage and reducing rescue frequency [65]. Our results on the stabilization of α-tubulin filaments and the increase in acetylated tubulin in K89R-expressing cells strongly support a protective response to the nocodazole insult. This implies that Lys-89 deSUMOylation might promote conditions in which the microtubule turnover rate is restrained, thereby maintaining microtubule stabilization in the presence of nocodazole. However, the increase in marker genes associated with microtubule stability, such as *MAP2, PCNT*, and *MAPT*, in both wild-type and K137R cells also denotes a role of Nor1 SUMOylation in regulating critical effectors of microtubule dynamics. Indeed, MAP2 and Tau proteins are known to bind microtubules, ensuring their protection from severing enzymes and depolymerization. Defects in their function are often associated with tauopathies or MAP2opathies, characterizing various neurodevelopmental and neurodegenerative conditions [20, 66]. Considering that Lys-89 and Lys-137 SUMOylation oppositely affect Nor1 transcriptional potential, our results suggest that disturbances in signaling pathways governing SUMO conjugation at either site could impair the proper regulation of microtubule critical regulators by Nor1, thereby affecting neuronal function. Several kinase pathways are known to contribute to the pathological phosphorylation of Tau, leading to neurological disorders [66–68]. Therefore, such kinase activation, which is required to coordinate Lys-137 SUMO2/3 conjugation and transcriptional repression of Nor1, might further impair MAPT expression and microtubule organization.

Our results revealed that impairment of Lys-89 SUMOylation led to increased levels of acetylated Lys-40 α-tubulin in neural cells treated with nocodazole, implying a role of Nor1 in regulating microtubule acetylation. Tubulin Lys-40 deacetylation in cells deficient in αTAT1 acetyltransferase has been associated with reduced resistance to nocodazole and mechanical stress, suggesting a crucial role for acetylation in microtubule longevity [46, 47, 69]. Furthermore, defects in tubulin acetylation have been linked to cognitive deficits and various neurodegenerative conditions [70, 71]. αTAT1 is considered the major, if not the sole, tubulin-acetylating enzyme in vivo [69, 72] and our findings that the ATAT1 gene and tubulin acetylation levels can be selectively regulated based on Nor1-specific SUMOylation argue for a critical, hence protective, role of Nor1 in responding to microtubule disturbances. Our results using fluorescence microscopy have also delineated the importance of Lys-40 acetylation in live cells subjected to nocodazole stress, whereby the K89R variant was more effective in maintaining α-tubulin content. However, given that these effects were partially blunted in the context of the acetyl-defective K40R or the acetyl-mimic K40Q α-tubulin variant, this indicates that besides acetylation, other tubulin turnover mechanisms might also support Nor1’s tubulin protection against depolymerization. Modifications such as ubiquitination and (de)tyrosination of tubulin have been implicated in modulating tubulin turnover and stabilization [73–75], thereby representing possible additional clues as to how Nor1 SUMOylation can regulate microtubule dynamics. Therefore, investigating whether such Nor1 effects on tubulin modifications primarily confer enhanced stability to de novo elongating microtubules or are essential for maintaining the structure of long-lived stable microtubules will certainly help in better understanding the intricate nature of Nor1 SUMOylation in responding to microtubule lattice damages.

## Concluding remarks

Our study has uncovered a novel mechanism by which Nor1 SUMOylation directly regulates microtubule dynamics in neuronal cells. The identification of Nor1 as a transcriptional regulator of genes involved in microtubule reorganization provides new insights into the hierarchical role of SUMOylation in fine-tuning neuronal gene expression networks and warrants further investigation of Nor1 as a potential therapeutic target in neurodegenerative diseases associated with microtubule dysfunction.

### Supplementary Information

Supplementary Material 1.

## Data Availability

Data sharing is not applicable. Materials used in the current study are available from the corresponding author on reasonable request.
