# Supplementary material for: SUMOylation of nuclear receptor Nor1/NR4A3 coordinates microtubule cytoskeletal dynamics and stability in neuronal cells
Source: Cell Biosci. 2024 Jul 13;14:91. doi: 10.1186/s13578-024-01273-x (PMC11245793; doi:10.1186/s13578-024-01273-x)
Supplement: Supplementary file 1 — Supplementary Material 1. [file 13578_2024_1273_MOESM1_ESM.pdf]

Fig. S1

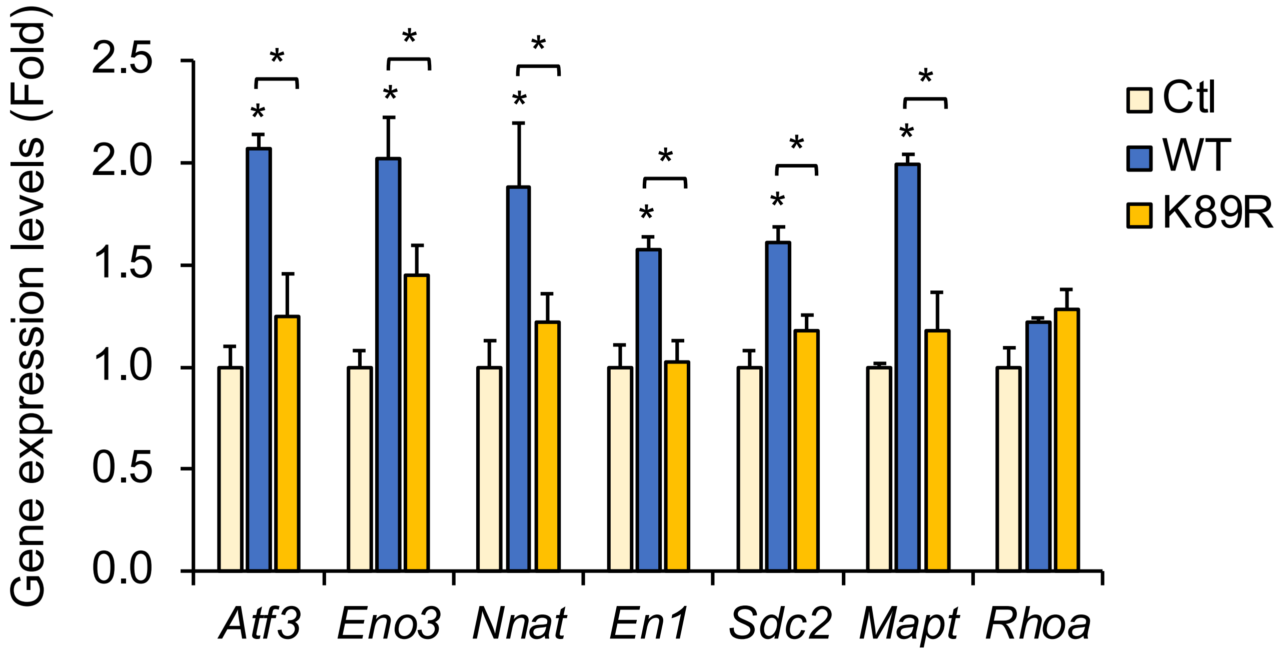

Figure S1. Nor1 SUMOylation at Lys-89 induces target gene expression in neuronal cells

qPCR analysis performed in mouse neuronal HT-22 cells transfected with wild-type or K89R mutated Nor1. Gene expression levels are derived from at least three separate experiments and normalized to RPLP0 expression. Results are expressed as fold response (mean ± SEM) relative to control cells (Ctrl) set to 1.0 for each gene. *, $P < 0.05$.

Fig. S2

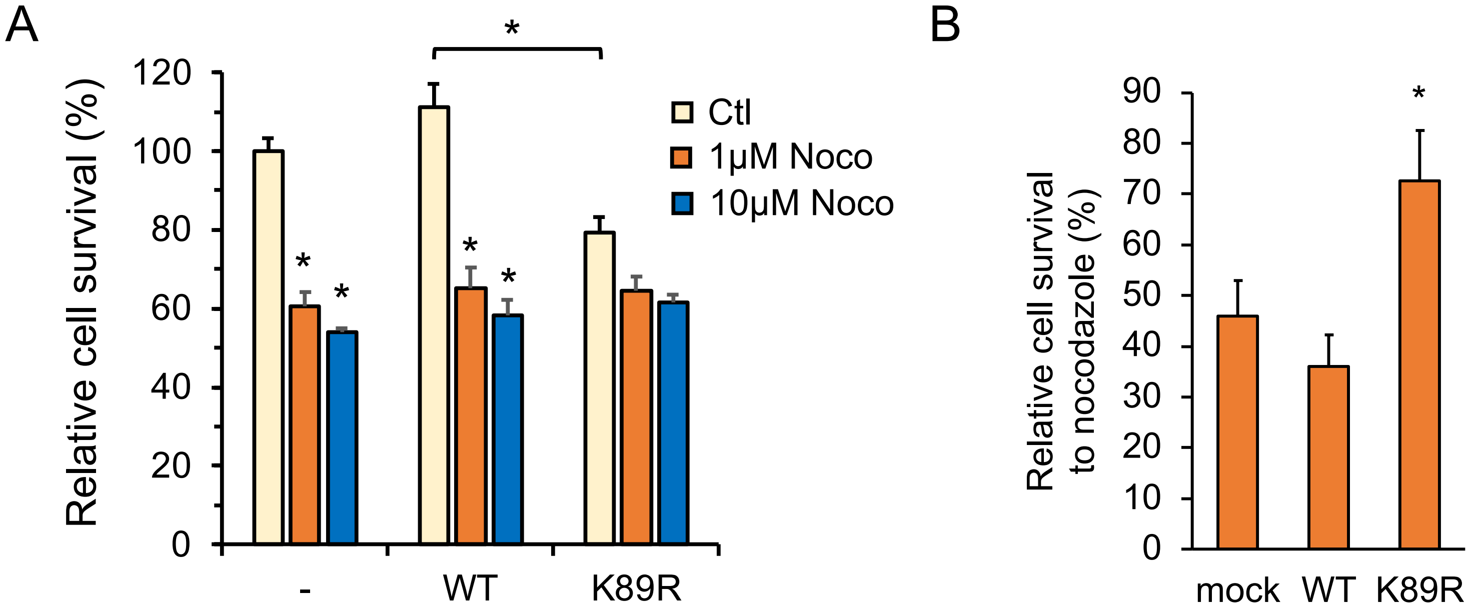

Figure S2. Nor1 Lys-89 SUMOylation affects neuronal cell survival to microtubule disorganization

(A) MTT cell viability assay was performed in Neuro-2A cells expressing wt or K89R Nor1. Cells were treated or not (vehicle) with 1 μM nocodazole for 6 hours. Values are presented as mean ± SEM of percent change compared to untreated control (-) cells set at 100%. Results are derived from at least three separate experiments. *, $P < 0.05$. **(B)** Same as in (A) except that SH-SY5Y cells stably expressing wt Nor1 or K89R mutated Nor1 were used and compared to mock-transfected cells. Results represent the mean ± SEM of percent change relative to each untreated cells. *, $P < 0.05$ vs mock cells.

Fig. S3

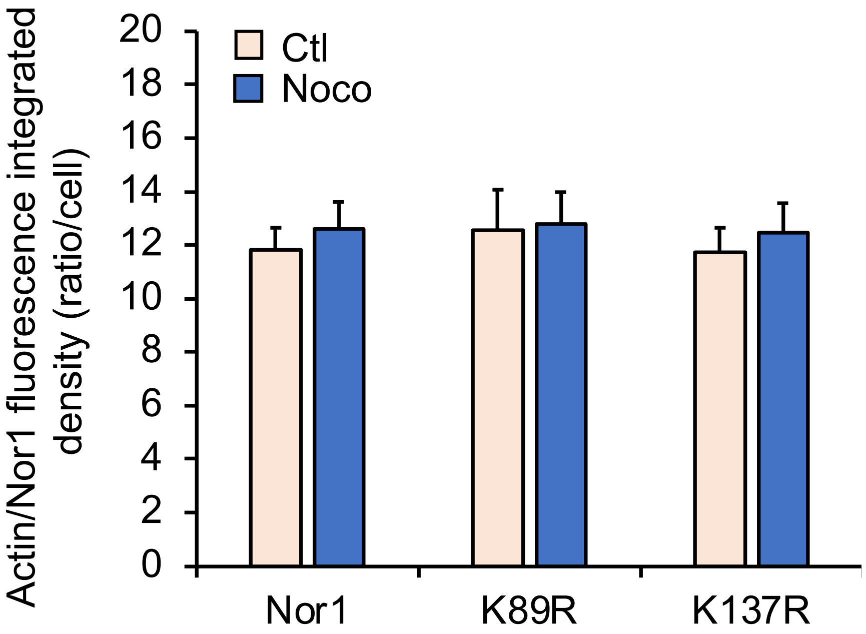

Figure S3. Nor1 SUMOylation does not affect cytoskeletal actin dynamics in neuronal cells

Mouse HT-22 neuronal cells were transfected with YFP fusions of WT, K89R or K137R Nor1 variant in the presence of mCherry-actin expression plasmid. Cells were then treated or not with 1μM nocodazole for 4hrs and vizualized in real-time by fluorescent microscopy. Quantitation of red (Cherry) fluorescence was determined and normalized to yellow (YFP) fluorescence for each respective cell using ImageJ. Mean fluorescence background values were subtracted in each group. Values are presented as mean ± SEM of actin to Nor1 ratios in untreated control (Ctl) and nocodazole-treated (Noco) cells derived from three independent experiments.
